# Effects of Olive Leaf (*Olea europaea*) Phenolic Extract on Zootechnical Parameters, Centesimal Composition, and Biochemical Parameters of Nile Tilapia (*Oreochromis niloticus*) Juveniles

**DOI:** 10.3390/ani15202935

**Published:** 2025-10-10

**Authors:** Thaise Dalferth Zancan, José María Monserrat, Vilásia Guimarães Martins, Marcelo Borges Tesser

**Affiliations:** 1 Institute of Oceanography, Federal University of Rio Grande, Av. Itália, km 8, Rio Grande 96203-900, Brazil; th4ise@gmail.com; 2 Marine Aquaculture Station, Federal University of Rio Grande, Rua do Hotel, 2, Rio Grande 96210-030, Brazil; josemmonserrat@gmail.com; 3 Laboratory of Nutrition of Aquatic Organisms, Rio Grande 96210-030, Brazil; 4 Institute of Biological Sciences, Federal University of Rio Grande, Av. Itália, km 8, Rio Grande 96203-900, Brazil; 5 Laboratory of Functional Biochemistry of Aquatic Organisms, Rio Grande 96210-030, Brazil; 6 School of Chemistry and Food Engineering, Federal University of Rio Grande, Av. Itália, km 8, Rio Grande 96203-900, Brazil; vilasiamartins@gmail.com; 7 Laboratory of Food Technology, Rio Grande 96203-900, Brazil

**Keywords:** aquaculture, fish nutrition, centesimal composition, antioxidant activity, biochemical parameters, lipid peroxidation

## Abstract

**Simple Summary:**

Fish farming plays a key role in producing healthy food and meeting the growing demand for animal protein. However, producers face challenges in maintaining fish health while reducing the use of synthetic additives. Olive leaves, a by-product of olive oil production, are rich in natural compounds that may improve fish health and product quality. In this study, we tested whether adding small amounts of olive leaf extract to the diet of Nile tilapia juveniles could improve their growth, body composition, and health indicators. The fish were fed for 46 days with diets containing different amounts of olive leaf extract. Fish growth was not affected, but the lowest amount tested improved the proportion of healthy nutrients in the fish’s body, balanced certain blood parameters, and reduced fat oxidation in the liver, which is linked to better health. These results suggest that olive leaf extract can be used as a natural feed additive to promote fish health and make aquaculture more sustainable, while also adding value to an agricultural by-product that would otherwise be discarded.

**Abstract:**

This study evaluated the effects of dietary olive leaf extract (OLE) on Nile tilapia (*Oreochromis niloticus*) juveniles, focusing on growth parameters, centesimal composition, biochemical responses, and lipid peroxidation. OLE was extracted with 60% ethanol (1:20 w/v). Five diets, containing 34% crude protein and 8% lipids, were tested: a control (0 g/kg) and four with increasing OLE levels (0.25, 0.5, 1, and 2 g/kg). The 46-day trial included 225 fish (0.56 ± 0.11 g) distributed in 15 tanks. Growth performance was not affected, except for a higher condition factor in OLE0.25. This dose also resulted in lower moisture and higher lipid content, while all OLE treatments increased crude protein in fish. Muscle glycogen decreased in all OLE-fed groups, and liver glycogen was reduced in OLE0.25. Plasma triglycerides decreased in OLE0.5 and OLE0.25, while total plasma protein was lower in OLE2.0. Liver triglycerides were lower in OLE0.25 and higher in OLE0.5, whereas glucose showed a glycemic peak in OLE2.0. Hepatic lipid peroxidation was reduced in OLE2.0. Overall, dietary OLE did not compromise the growth performance of Nile tilapia, and 0.25 g/kg promoted beneficial effects on centesimal composition, biochemical parameters, and lipid peroxidation, highlighting its potential as a functional ingredient in aquafeeds.

## 1. Introduction

Aquaculture is one of the fastest-growing animal protein production sectors worldwide and has played a functioning role in food security and economic development in recent years [1]. In Brazil, this sector has gained prominence, particularly through the production of Nile tilapia (*Oreochromis niloticus*), one of the most widely farmed fish species worldwide [2]. Brazil ranks fourth among the largest producers [3] owing to its favorable characteristics, such as rapid growth, stress resistance, and broad commercial acceptance [4]. However, intensive growth of aquaculture poses significant challenges, such as stress resulting from high stocking densities, emergence of diseases, and the need for nutritional strategies that sustainably promote fish health and performance [5,6,7,8,9].

Over the years, the use of antibiotics has become routine practice in aquaculture to control bacterial pathogens and enhance fish performance [6,10]. However, their indiscriminate use has raised concerns such as environmental imbalance, the development of antibiotic-resistant strains, and the presence of residues in aquaculture systems [1,6,10]. Therefore, it is essential to seek safe, effective, and sustainable alternatives for promoting the health and growth of aquatic organisms. Among these, plant-derived bioactive compounds, particularly phenolics, are promising functional additives [9,11,12,13,14,15,16]. The phenolic compounds present in these extracts exhibit antioxidant, anti-inflammatory, antimicrobial, and immunomodulatory properties [5,12,13,16]. These compounds can enhance fish resistance to stress, stimulate the immune system, and modulate the expression of genes related to antioxidant and immune responses [12,17].

In this context, the valorization of agro-industrial by-products and wastes such as olive leaves through the extraction of phenolic compounds represents a sustainable strategy for adding value to the production chain by transforming waste into new functional ingredients [13,14,18]. Olive leaves (*Olea europaea*) constitute a by-product of olive cultivation resulting from pruning and can represent up to 10% of the weight of olives harvested for oil extraction [19]. They are a rich source of bioactive compounds, particularly phenolics, that exhibit various health benefits and functional applications [14,18,19,20]. Among these compounds, terpenes, such as secoiridoids, are restricted to the *Oleaceae* family and are predominant in olive leaves, whereas the phenolic compound oleuropein is the main molecule responsible for the antioxidant and antibacterial activities of olive leaf extracts [15,19,20]. Furthermore, olive leaves are rich in flavonoids and simple phenols, such as tyrosol and hydroxytyrosol, which contribute to combating oxidative stress and modulating inflammatory processes [14,19].

The chemical composition of olive leaves can vary according to factors, such as olive variety, geographical location, climatic conditions, plant maturation stage, harvest period, and drying and extraction methods [19,21]. These factors influence the concentrations of bioactive compounds, thereby affecting their biological activity [22]. The form of phenolic compounds present within the plant matrix also affects their solubility, bioavailability, and extraction efficiency [22]. Conventional solid–liquid extraction techniques using hydro-organic solvents have been widely employed to maximize the recovery of these compounds [14,15,23,24,25]. However, owing to toxicological and environmental concerns, as well as food and nutraceutical applications, the use of organic solvents is often limited to ethanol [14].

Olive leaf extract (OLE) has been studied as a natural alternative to antibiotics and has been shown to improve the physiological and immunological responses of fish [13,15,26]. Although many studies have reported the positive effects of OLE on fish nutrition, the efficacy of these compounds in fish nutrition still requires more in-depth research, particularly regarding their effects on zootechnical responses, body composition, carbohydrate and lipid metabolism, and other biochemical parameters. Furthermore, inconsistent results across different species and dosages highlight the need for further investigations. Thus, the objective of this study was to obtain a phenolic extract from olive leaves and evaluate its inclusion in the diets of Nile tilapia (*O. niloticus*) juveniles and its effects on the growth parameters, zootechnical indices, centesimal composition, and biochemical parameters of the fish.

## 2. Materials and Methods

### 2.1. Ethical Approval

The experiment was conducted at the Marine Aquaculture Station (EMA) of the Federal University of Rio Grande–FURG, located at Cassino, Rio Grande, RS (32°12′ S 52°10 W), from April to June 2024, for a total of 46 days. All procedures involving live animals strictly followed the guidelines of FURG’s Animal Use Ethics Committee (CEUA) under protocol no. 23116.018777/2023-23.

### 2.2. Extraction of Olive Leaf Extract (OLE)

**Figure 1:**
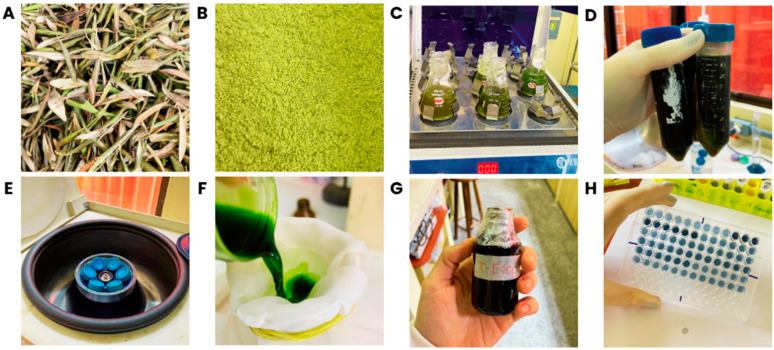
(**A**) Fresh olive leaves. (**B**) Ground and sieved olive leaves. (**C**) Extraction of phenolic compounds using different solvents in an orbital shaker. (**D**) Extracts obtained after shaking, transferred to Falcon tubes for centrifugation. (**E**) Centrifugation of the extracts. (**F**) Collection of the supernatants. (**G**) Extracts stored in amber flasks. (**H**) Quantification analyses of phenolic compounds.

Olive leaves (*O. europaea*) were donated by the Azeite Batalha factory (Pinheiro Machado, RS, Brazil) (Figure 1A). After washing under running water, leaves were dried at 45 °C for 12 h. The dried material was ground in a blender and sieved through a mesh with a 0.6 mm aperture (mesh 28), resulting in olive leaf powder (Figure 1B).

OLE was obtained using conventional extraction following the methodology proposed by Santos and Martins [24] (Figure 1C–G). Four solvents were tested for the extraction of phenolic compounds from olive leaf powder: distilled water (DA), 30% ethanol (E30), 60% ethanol (E60), and 100% ethanol (E100), in the proportion of 1:20 (*w*/*v*). The mixtures were stirred in an orbital shaker (TE-420, Tecnal, Piracicaba, Brazil) at 150 rpm for 60 min at room temperature (25 ± 1 °C) and centrifuged at 8694× *g* (MPW Med. Instruments, MPW-350, Warsaw, Poland) for 10 min at room temperature (25 ± 1 °C). Supernatants were collected and stored in amber flasks under refrigeration (4 ± 1 °C) until analysis.

The polyphenol content (TPC) and total flavonoid content (TFC) of the extracts were analyzed (Figure 1H). TPC was determined using the Folin–Ciocalteu method, according to Santos et al. [23], using a standard gallic acid curve. TFC was quantified using aluminum chloride (AlCl_3_) as a complexing agent, according to Santos and Martins [24], using a standard quercetin curve. OLE, with the highest TPC and TFC, was selected for inclusion in the experimental diets.

### 2.3. Experimental Diets

A control diet without the inclusion of OLE (OLE_0_) was formulated to contain 34% crude protein and 8% total lipids, serving as the basis for the creation of four other experimental diets with increasing levels of OLE inclusion: 0.25 g/kg (OLE_0.25_), 0.5 g/kg (OLE_0.5_), 1 g/kg (OLE_1.0_), and 2 g/kg (OLE_2.0_) (Table 1). The OLE used (E60) was dried to a final concentration of 12.27 mg/mL (dry matter) and added to the diets at the desired inclusion levels. All diets were supplemented with vitamin and mineral premix according to the manufacturer’s recommendations to meet the nutritional requirements of the species used.

The process of making the feed involved manual homogenization of all dry ingredients, followed by the addition of oil, water, and OLE. Next, the mixture was pelleted using a meat grinder to obtain the pellets, which were dried at 45 °C for 12 h. After drying, the diets were packed in plastic bags and stored at −18 °C until use. The diets were analyzed for centesimal composition, including moisture, ash, crude protein, and crude fiber, following the protocols of the AOAC [27]. The total lipid content was determined using the method described by Bligh and Dyer [28]. Non-nitrogenous extracts (NNE) were calculated by subtracting the moisture, ash, crude protein, crude fiber, and total lipids contents from 100, as described by Rodrigues [29]. All data are presented in Table 1.

### 2.4. Experimental Trial

Nile tilapia (*O. niloticus*) juveniles, sexually reversed, were obtained through a donation from the Aquaculture Laboratory of the Federal University of Pelotas (UFPEL). The fish were transferred to the EMA, where they underwent a seven-day acclimatization period to adapt to the laboratory conditions before the experiment began. During this period, the fish were kept in an 850 L tank, provided with constant aeration, and the salinity of the water was gradually increased until it reached approximately 10 ‰, according to the recommendations of Dawood et al. [30]. The fish were fed three times a day (9 a.m., 12 p.m., and 3 p.m.) with an extruded commercial diet (Wean Prime 40, Bernaqua) until apparent satiety.

**Figure 2:**
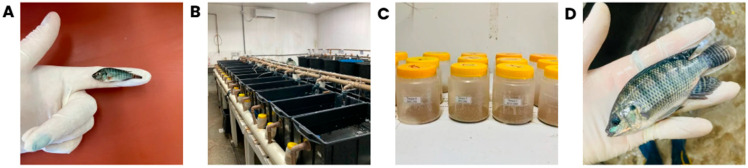
(**A**) Juvenile Nile tilapia at the start of the experimental trial. (**B**) Arrangement of tanks in the recirculating aquaculture system. (**C**) Containers used for diet storage and daily weighing to monitor feed intake. (**D**) Juvenile Nile tilapia at the end of the experimental trial.

After the acclimatization period, 225 fish (0.56 ± 0.11 g) were distributed in 15 rectangular tanks (70 L), maintaining a stocking density of 15 fish per tank (Figure 1A and Figure 2A). The tanks were arranged in a water recirculation system, containing a biofilter and mechanical filter, and supplied with brackish water. The experiment was conducted using a completely randomized design, consisting of five treatments, each applied to three tanks. The photoperiod was maintained at 12 h light and 12 h dark. Daily feed intake per tank was calculated by measuring the difference in the weight of the feed container before the first meal and after the last meal of the day (Figure 2C).

#### Water Quality

The physicochemical parameters of the water were monitored daily. Temperature and dissolved oxygen were measured twice daily (in the early morning and late afternoon) using a multiparametric probe (YSI, 550A, Yellow Springs, OH, USA). The water temperature was adjusted using submerged thermostatic heaters. Total ammonia, nitrite, and nitrate concentrations were determined according to methods proposed by UNESCO [31], Aminot and Chaussepied [32], and García-Robledo et al. [33], respectively. Total alkalinity was determined according to the protocol established by APHA [34] and adjusted with sodium bicarbonate, as needed. The pH was measured using a digital pH meter (Mettler Toledo, FiveEasy, Columbus, OH, USA), and the salinity was determined using a portable refractometer. Each tank is equipped with an individual aerator. The water renewal rate was maintained at 2 L/min per tank, ensuring the removal of metabolites and maintaining the water quality.

### 2.5. Data Collection

At the end of the experimental period, the fish were fasted for 24 h to reduce the gastrointestinal content. Fish were anesthetized using eugenol solution (50 mg/L) [35], and individual morphometric measurements were recorded as standard length (SL), total length (TL), and final body weight (FBW).

Blood samples were collected from three fish per tank by puncturing the caudal vein using heparinized syringes. Plasma was obtained after centrifugation of blood at 637× *g* (Solab, SL-703, Piracicaba, Brazil) for 10 min at 4 °C and stored at −80 °C for subsequent analysis. After blood collection, the fish were euthanized by eugenol overdose (250 mg/L) and dissected for liver and viscera collection to determine zootechnical indices. Measurements included the weights of the liver, viscera, and carcass, and the length of the intestine.

### 2.6. Zootechnical Performance

#### 2.6.1. Growth Parameters

Growth parameters, including weight gain (WG), daily weight gain (DWG), specific growth rate (SGR), survival rate (SR), feed conversion rate (FCR), and condition factor (CF) were calculated using the following equations:

WG (g) = final body weight (g) − initial body weight (g)(1)

DWG (g) = WG/experimental period (days)(2)

SGR (%/day) = {[ln (FBW) − ln (IBW)]/experimental period (days)} × 100(3)

SR (%) = (final number of fish/initial number of fish) × 100(4)

FCR (g/g) = feed intake (g)/WG(5)

CF = [FBW/(TL)^3^] × 100 (6)

#### 2.6.2. Zootechnical Indices

Based on the data of liver, viscera and carcass weight, and intestinal length, the hepatosomatic index (HSI), viscerosomatic index (VSI), carcass yield (CY), and intestinal quotient (IQ) were calculated using the following equations:

HSI (%) = (liver weight (g)/FBW) × 100(7)

VSI (%) = (viscera weight (g)/FBW) × 100(8)

CY (%) = (carcass weight (g)/FBW) × 100(9)

IQ = (intestinal lenght (cm)/SL (cm)) × 100(10)

### 2.7. Centesimal Composition and Nutrient Deposition

Three fish from each tank were dried at 45 °C for approximately 12 h until they reached constant weight. Subsequently, the fish were macerated to obtain samples for further analysis. The same procedure was performed on an initial sample of the fish to determine the initial body composition.

Moisture, ash, and crude protein contents were determined according to the protocols described by AOAC [27]. The crude protein content was determined using the micro-Kjeldahl method, employing a conversion factor of 6.25 for the conversion of total nitrogen into crude protein. Total lipids were determined using a cold extraction method, according to the methodology described by Bligh and Dyer [28]. Nutrient deposition rates (NDR) were calculated using the following equation:

NDR (g) = [FWB × (% FBN/100)] − [IWB × (% IBN/100)](11)

where FWB and IWB represent the final and initial body weights (g), respectively, and FBN and IBN correspond to the final and initial body nutrient contents (crude protein or lipids), respectively.

### 2.8. Biochemical Analyses

#### 2.8.1. Tissue Homogenization

Liver and muscle tissue samples were homogenized (1:5 *w*/*v*) in Tris-HCl buffer solution (100 mM, pH 7.78) containing EDTA (2 mM) and Mg2+ (5 mM) [36]. Crude homogenate was used to determine the hepatic and muscle glycogen levels. The homogenates were centrifuged at 20.000× *g* (Solab, SL-703, Piracicaba, Brazil) for 10 min at 4 °C to separate the supernatant, which was collected and stored at −80 °C for subsequent analyses.

#### 2.8.2. Muscle and Liver Glycogen

The muscle and liver glycogen contents of the fish were quantified using the phenol-sulfuric acid colorimetric method according to the protocol described by Schaubroeck, Leitner, and Perry [37]. This method is based on the formation of a colorimetric complex, the intensity of which is directly proportional to the concentration of glucose released during glycogen hydrolysis. For extraction, 180 µL of the homogenate (1:5 *w*/*v*) was incubated with 20 µL of sodium hydroxide 5 M and heated to 100 °C for 30 min. Glycogen was precipitated after the addition of 50 µL 9.5% sodium sulfate and 600 µL absolute ethanol, followed by centrifugation at 5000× *g* (Solab, SL-703, Piracicaba, Brazil) for 5 min to isolate the glycogen pellet. The pellet was resuspended in 500 µL of distilled water, and 50 µL of this solution was used for the colorimetric reaction. The reaction was initiated by adding 300 µL of concentrated sulfuric acid and 45 µL of 5% phenol solution. After 30 min of incubation at room temperature (25 ± 1 °C), absorbance was measured at 488 nm. Glycogen quantification was performed using a glucose standard curve, and the results are expressed in milligrams of glucose equivalents (GE) per gram of tissue (mg GE/g).

#### 2.8.3. Plasma and Liver Biochemical Parameters

Plasma and hepatic concentrations of glucose, cholesterol, triglycerides, and total protein were quantified using colorimetric enzymatic assays with commercial kits from Labtest Diagnóstica S.A. (Lagoa Santa, Brazil). Assays were performed according to the manufacturer’s instructions, using the following kits. For the analysis of hepatic biochemical parameters, the results were adjusted by considering the dilution of the liver samples during the tissue homogenization stage.

#### 2.8.4. Lipid Peroxidation

Quantification of lipid peroxidation levels (TBARS) in the muscle and liver was performed according to the methodology proposed by Oakes and Van der Kraak [38], which quantifies oxidative stress by measuring malondialdehyde (MDA), a by-product of lipid peroxidation. MDA reacts with thiobarbituric acid (TBA) under high-temperature and acidic conditions to form a fluorescent chromogen. For analysis, homogenized liver (50 µL) and muscle (100 µL) samples were incubated with 20 µL BHT solution (67 µM), 150 μL acetic acid solution (20%), 150 μL TBA solution (0.8%), 50 μL ultrapure water, and 20 μL sodium dodecyl sulfate (SDS) solution (8.1%). The mixture was heated in a water bath at 95 °C for 60 min. After cooling, 100 μL ultrapure water and 500 μL n-butanol were added to the final solution. The samples were centrifuged at 917× *g* (Solab, SL-703, Piracicaba, Brazil) for 10 min at 15 °C. Supernatants were collected, and fluorescence was determined using a spectrofluorometer (excitation: 515 nm; emission: 553 nm). Lipid peroxidation levels were expressed as nanomoles of MDA per milligram of tissue using a standard tetramethoxypropane (TMP) curve.

### 2.9. Statistical Analysis

Statistical analysis of the data was performed using R software (version 4.2.1, Posit Software, PCB, Boston, MA, USA). The normality of the data and the homogeneity of variance were evaluated. After verifying these assumptions, the data were subjected to unidirectional analysis of variance (ANOVA). In the presence of statistically significant differences between treatments (*p* < 0.05), means were compared using Tukey’s post hoc test. In cases where the premises of ANOVA were not met, the data were analyzed using the non-parametric Kruskall–Wallis test. In the case of statistically significant differences (*p* < 0.05), the means were compared using Dunn’s post hoc test. All data are presented as mean ± standard deviation.

## 3. Results

### 3.1. Olive Leaf Extract (OLE)

**Figure 3:**
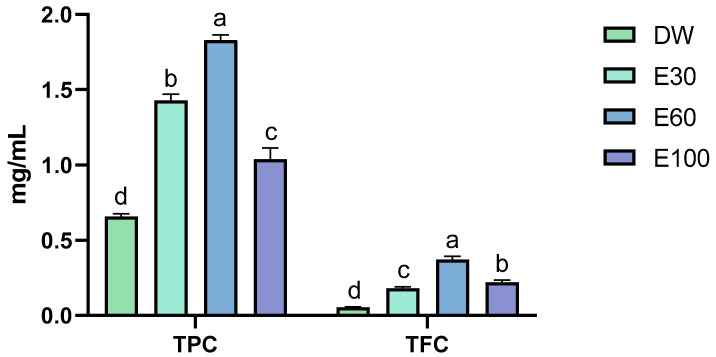
TPC and TFC of the extracts obtained with different solvents for determining the OLE used in the experimental diets. Data are expressed as mean ± standard deviation (*n* = 3). Values with different letters indicate significant differences between the treatments (*p* < 0.05).

The TPC and TFC values of the extracts obtained using the different solvents are shown in Figure 3. The highest levels of TPC (1.82 ± 0.03 mg/mL) and TFC (0.37 ± 0.02) were observed in the extract obtained with E60, which was selected for the formulation of experimental diets.

### 3.2. Water Quality

Throughout the experimental period, the physicochemical parameters of the water remained within the limits considered adequate for Nile tilapia [39]: dissolved oxygen 6.39 ± 0.94 mg/L, temperature 26.60 ± 1.20 °C, pH 7.81 ± 0.33, total ammonia 0.23 ± 0.38, nitrite 6.84 ± 6.14 mg/L, nitrate 6.55 ± 6.77 mg/L, salinity 10.91 ± 1.38 ‰, and alkalinity 123.19 ± 37.05 mg CaCO_3_/L.

### 3.3. Zootechnical Performance

#### 3.3.1. Growth Parameters

The growth parameters of the fish are listed in Table 2. The groups did not show significant differences (*p* > 0.05) in FW, WG, DWG, SGR, SR, or FCR. However, CF was influenced by the treatments (*p* < 0.05), in which there was a reduction in CF with an increase in the dose of OLE in the diets, with the highest value observed in the OLE_0.25_ group and the lowest in the OLE_2.0_ group.

#### 3.3.2. Zootechnical Indices

The zootechnical indices of fish are listed in Table 3. The groups did not demonstrate statistically significant differences (*p* > 0.05) in HSI, VSI, CY, or IQ.

### 3.4. Centesimal Composition and Nutrient Deposition

Data on centesimal composition and nutrient deposition rates of the fish are presented in Table 4. No significant differences (*p* > 0.05) were observed in ash content, protein or lipid deposition between the groups. A reduction in moisture content was observed in fish fed the OLE_0.25_ diet (*p* < 0.05). The crude protein content of the fish increased in the groups fed diets containing OLE (*p* < 0.05). Lipid content increased to a maximum in the OLE_0.25_ group (*p* < 0.05), whereas a significant lower levels were observed in the OLE_0_ and OLE_2.0_ groups (*p* < 0.05).

### 3.5. Biochemical Analyses

#### 3.5.1. Muscle and Liver Glycogen

Muscle and liver glycogen levels are listed in Table 5. There was a significant reduction in muscle glycogen in all groups that received diets containing OLE (*p* < 0.05), while liver glycogen was reduced in the OLE_0.25_ group, followed by the OLE_1.0_ and OLE_2.0_ groups, with an increase observed only in the OLE_0.5_ group (*p* < 0.05), which showed levels similar to those of the control group (*p* < 0.05).

#### 3.5.2. Plasma and Liver Biochemical Parameters

The plasma and hepatic parameters of the fish are listed in Table 5. Regarding the plasmatic parameters, glucose and cholesterol levels did not differ significantly between the groups (*p* > 0.05). Triglyceride levels were higher in the OLE_1.0_ group (*p* < 0.05). The total protein levels were lower in the OLE_2.0_ group than in the OLE_1.0_ group (*p* < 0.05). Liver cholesterol and total protein levels were not significantly different between groups (*p* > 0.05). There was a peak of glucose in the OLE_2.0_ group compared to that in the other treatments (*p* < 0.05). Triglycerides showed lower levels in the OLE_0.25_ group when compared with the OLE_0.5_ group (*p* < 0.05).

#### 3.5.3. Lipid Peroxidation

**Figure 4:**
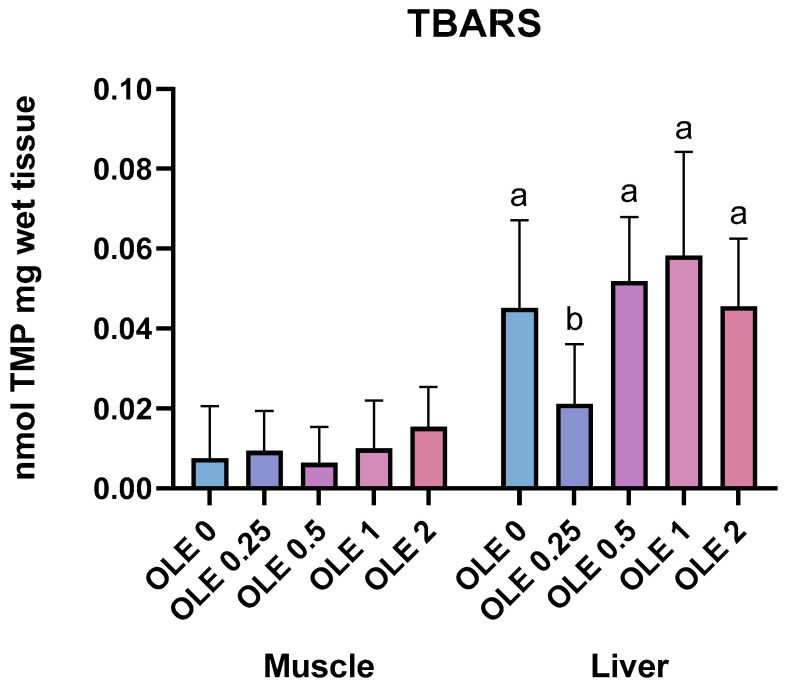
Thiobarbituric acid reactive substances in the muscle and liver of Nile tilapia juveniles fed experimental diets. Values with different letters indicate significant differences between the treatments (*p* < 0.05).

Lipid peroxidation (TBARS) in the muscle and liver of fish is shown in Figure 4. Muscle TBARS levels were not affected by diet (*p* > 0.05). On the other hand, in the liver, a reduction was observed in the OLE_0.25_ group (*p* < 0.05).

## 4. Discussion

Olive leaf extracts have attracted attention as a sustainable and effective approach to improve stress resistance and growth, as well as biochemical and immune parameters in fish [13,15,26,40,41,42,43,44,45]. In the present study, OLE supplementation did not affect fish growth or zootechnical indices, with the exception factor (CF). CF is a morphometric index used to assess the health and well-being of a fish, based on its weight and length [46]. In our study, there was a reduction in CF with an increase in the dose of OLE in the diet, which was more evident at the highest level of OLE inclusion (2 g/kg). This reduction can be explained by the hormetic response induced by phenolic compounds; that is, at low concentrations, polyphenols can have beneficial effects on fish growth and metabolism; however, high doses can act as anti-nutritional compounds or trigger pro-oxidant effects, thereby impacting fish development and well-being [47]. Thus, a hermetic response should explain why the administration of high doses of OLE to fish diets induces oxidative stress [13,41,42,43]. The absence of changes in CF observed by Sokooti et al. [15] with the inclusion of 200 and 400 mg OLE/kg of feed was consistent with the results of the present study, in which lower doses did not influence this parameter when compared to the control treatment (Table 2).

While low doses of OLE may be beneficial, high concentrations may cause adverse effects, such as interference with nutrient absorption or oxidative stress, affecting the body composition of fish [47]. Hussain et al. [48] observed an inverse relationship between the level of polyphenols in the diet of fish and their centesimal composition, in which the lowest dose (200 mg/kg) resulted in the highest protein content, whereas the highest dose (800 mg/kg) led to a reduced protein content and increased lipid content, indicating the beneficial effect of smaller doses. In our study, the ash content and protein and lipid deposition in fish were not affected by diets containing OLE. However, a reduction in moisture was observed at the lowest level of OLE inclusion (0.25 g/kg), while this group had the highest lipid content. The protein content of the fish increased in the OLE-treated groups. Some studies have suggested that the phenolic compounds present in plant extracts may favor the absorption and use of nutrients [48,49]. However, studies that evaluated the centesimal composition of fish after OLE supplementation found no significant differences compared with the control group [15,26]. Regarding lipids, the increase observed only in the group fed the lowest OLE inclusion suggested that higher doses may have reduced the absorption or accumulation of fats. Jiménez-Sánchez et al. [50] demonstrated that olive leaf extracts are effective in reducing lipid accumulation in hypertrophic and insulin-resistant adipocytes, an effect associated with AMPK activation by phosphorylation. AMP-activated protein kinase (AMPK) acts as a central sensor of cellular energy status, detects changes in energy and nutrient levels, and is activated when cellular ATP levels decrease. When activated, it phosphorylates several downstream targets involved in cellular metabolism, promoting a coordinated response that potentiates ATP-generating catabolic pathways, such as fatty acid oxidation, while inhibiting ATP-consuming anabolic processes, such as lipid synthesis [51,52,53]. The reduction in lipid content in the centesimal composition of the fish in our study was only observed at higher inclusion levels of OLE, corroborating these findings. In contrast, Sokooti et al. [15] reported a decrease in carcass lipid content at the lowest tested dose of OLE (200 mg/kg), which was comparable to the lowest dose used in our experiment. These findings suggest that in our case, a higher concentration of OLE was required to produce a similar lipid-lowering effect. Naz et al. [26] found no differences in the body composition of fish with OLE inclusions of 0–2%. However, the influence of OLE on the body composition of fish remains unclear.

Glucose and glycogen metabolism are the primary metabolic pathways responsible for maintaining the energy balance in response to various environmental stimuli [51]. Glycogen is a branched polymer that acts as a glucose reserve in living organisms and is mobilized according to energy needs [54,55]. Under food deprivation conditions, glycogen depletion occurs through glycogenolysis or glucose synthesis from other molecules via alternative pathways, such as gluconeogenesis [54]. In our study, we observed a reduction in muscle glycogen levels in fish with the inclusion of OLE in their diet. This observation may be related to the presence of phenolic compounds in OLE, especially oleuropein, which can activate the phosphorylation of AMP-activated protein kinase (AMPK) and promoting increased glucose uptake, a catabolic pathway that generates ATP [50,56]. AMPK plays an important role in carbohydrate metabolism [51]. Once activated, AMPK favors fatty acid oxidation and tissue use of glucose, while inhibiting glycogen synthesis and storage [52]. These mechanisms may have contributed to the greater mobilization of muscle glycogen to meet energy demands, possibly due to the mild metabolic stress induced by OLE in diets. In fact, a review by Esteras and Abramov [57] highlighted that the transcriptional factor Nrf2, which drives the upregulation of antioxidant genes at the same time, induces genes associated with ATP generation pathways, such as glycolysis, Krebs cycle, and oxidative phosphorylation.

The liver plays a central role in carbohydrate metabolism, maintaining glycemic homeostasis through the uptake and storage of glucose after meals and its release during fasting [54]. Although the control treatment (OLE_0_) did not differ from the others, we observed that the group that received 0.25 g/kg of OLE had the lowest hepatic glycogen level, while the dose of 0.5 g/kg resulted in the highest level. In contrast, treatments with higher inclusion levels (1 and 2 g/kg) had intermediate values. These results suggest that the lower dose may have intensified AMPK activation or elicited an acute response to phenolic compounds, leading to mobilization of hepatic glycogen stores. On the other hand, the dose of 0.5 g/kg may have reached an equilibrium point, characteristic of hormesis, at which phenolic compounds improved glucose uptake and hepatic glycogen synthesis. Oleuropein, the main bioactive compound in OLE, has been associated with positive anabolic modulation of glucose metabolism [58], which reinforces the hypothesis of distinct metabolic adaptations according to the dose administered.

Despite these changes in glycogen stores, plasma glucose levels remained stable in all groups, indicating that glycemic homeostasis was preserved. OLE is known for its hypoglycemic effects, which are attributed to its antioxidant potential, as well as the regulation of hyperglycemia through improved glucose-induced insulin release and increased peripheral glucose uptake [59]. Oleuropein exerts beneficial effects on glucose metabolism by promoting glycolysis and reducing gluconeogenesis, contributing to the regulation of glycemic homeostasis [60]. This result may indicate that OLE does not affect the glycemic balance of fish, even though it promotes glycogen mobilization or accumulation in different tissues. It is worth noting that, while liver glucose remained stable in most treatments, an increase was observed in the highest dose of OLE inclusion (2 g/kg). Although high doses of phenolic compounds may exert pro-oxidant effects and increase the production of reactive oxygen species [61,62], the resulting impact on metabolism is dose dependent. According to Esteras and Abramov [57], moderate pro-oxidant conditions can stimulate catabolic processes rather than suppressing metabolism, highlighting the complex and adaptive nature of cellular responses to oxidative stress. Glucose levels are considered to be indicators of stress [15,63]. Similarly, Zemheri-Navruz et al. [44] reported a glycemic spike in the serum of fish fed diets containing 0.5% and 1% OLE, suggesting that there may be a saturation point at which OLE begins to negatively affect certain metabolic pathways.

Lipids are among the main nutrients responsible for providing energy and essential fatty acids for fish growth and metabolism. In addition, they participate in the transport of fat-soluble vitamins and act as hormone precursors [64]. Lipid metabolism plays a fundamental role in the maintenance of energy homeostasis in the body. However, elevated circulating levels of lipids such as cholesterol and triglycerides may indicate a lipid metabolism disorder [65]. In our study, the plasma and liver cholesterol levels of fish were not affected by the inclusion of OLE in their diets. Similar results were reported by Baba et al. [63], who observed no changes in serum cholesterol levels of rainbow trout (*Oncorhynchus mykiss*) fed OLE-containing diets. However, other studies have also reported conflicting results. Fazio et al. [40] reported a progressive reduction in cholesterol levels with increased inclusion of OLE in the diet of Nile tilapia (*O. niloticus*). Similarly, Sokooti et al. [15] observed decreased cholesterol levels in carp (*Cyprinus carpio*) fed OLE-containing diets, whereas Assar et al. [13] attributed the reduction in cholesterol in fish to greater mobilization of fatty acids from adipose tissue to the liver. Zemheri-Navruz et al. [44] observed an increase in cholesterol levels in carp (*C. carpio*) fed diets containing 1% OLE, suggesting that higher doses of the extract may negatively affect fish health. Unlike triglycerides, which are directly affected by alterations in fatty acid uptake, synthesis, and mobilization [66], cholesterol is regulated by homeostatic mechanisms involving the regulation of 3-hydroxy-3-methylglutaryl coenzyme A reductase (HMGCR), uptake of cholesterol from systemic circulation via low-density lipoprotein (LDLR) receptors, transport of excess cholesterol to the liver via high-density lipoprotein (HDL) receptors, metabolism, and excretion [67,68], making cholesterol less responsive to acute dietary changes.

Triglycerides are important indicators of lipid metabolism in fishes. In the liver and adipose tissues, surplus energy is stored in the form of triglycerides via the fatty acid synthesis pathway [53]. In our study, a reduction in plasma triglyceride levels was observed in the groups fed 0.25 and 0.5 g/kg of OLE, as well as in liver triglycerides in the group that received 0.25 g/kg of OLE. These effects may be related to the ability of olive leaf extracts to reduce lipid accumulation, as demonstrated by Jiménez-Sánchez et al. [50], who reported that this action is associated with AMPK activation. In agreement with our findings, Sokooti et al. [15] also reported a decrease in triglyceride levels in carp (*C. carpio*) fed a diet supplemented with 200 mg/kg OLE, suggesting a lipid-lowering effect associated with OLE.

The hypolipidemic and antihyperlipidemic effects of olive leaf extract and oleuropein have been demonstrated in rat studies [60,69,70], mice [71,72], and rabbits [73]. In mice, it has been observed the administration of 100 mg/kg oleuropein may reduce serum and hepatic triglyceride levels [71]. At low doses, oleuropein acts as a ligand for peroxisome proliferator-activated receptor α (PPARα), promoting the upregulation of several genes that encode enzymes involved in the uptake, transport, and oxidation of fatty acids, which contribute to the reduction in serum triglyceride levels induced by this compound [71]. In addition, phenolic compounds such as oleuropein can activate AMPK, stimulate fatty acid oxidation, inhibit lipogenesis [50,56], and promote autophagy [72], which may explain the reduction in plasma triglycerides at these doses.

This effect has been previously described, demonstrating that polyphenols activate AMPK, which inhibits the activity of lipogenic enzymes, such as acetyl-CoA carboxylase (ACC), an important regulatory site in fatty acid synthesis and oxidation pathways [51,53], promoting lipid catabolism and, consequently, reducing fat deposition. Activation of AMPK can reduce triglyceride accumulation in hepatocytes, where ACC1 inhibits fatty acid synthesis [53]. Although in the present study we did not directly assess AMPK activity of ACC expression, it is possible that such pathways are involved in the lower hepatic triglyceride levels observed in the OLE_0.25_ group in respect to the OLE_0.5_ group (Table 5). Baba et al. [63] reported a significant decrease in some groups fed diets containing OLE (0.5 and 1%) on diets for rainbow trout (*O. mykiss*). Fazio et al. [40] reported a reduction in triglyceride level with increased inclusion of OLE in diets for Nile tilapia (*O. niloticus*), attributing the use of fat as an energy source. Assar et al. [13] observed that triglyceride levels were lower in fish fed a diet containing 0.1% OLE, attributing this reduction in response to increased fatty acid influx from adipose tissue to the liver. High doses of OLE have been linked to oxidative stress and hepatotoxicity in fish [41,42]. In our study, the increase in triglyceride levels at the highest doses of OLE (1 and 2 g/kg) may indicate a loss of the beneficial effects of phenolic compounds, possibly by the induction of oxidative stress.

Total plasma protein is widely used as an indicator of humoral immunity and innate immune response in fish [74]. Elevations in this parameter, including albumins and globulins, have often been suggested as signs of enhanced immune responses, and are interpreted as evidence of a stronger immune system [26,41,75]. Albumin is the most abundant plasma protein in animals and is involved in the transport of various substances, in addition to participating in the regulation of blood volume and the maintenance of oncotic pressure in body fluids [76]. Globulins α and β are acute-phase proteins responsive to inflammation, whereas γ-globulins consist mainly of immunoglobulins involved in humoral immunity in fish [76]. In our study, an increase in the total plasma protein levels of fish was observed up to the inclusion of 1 g/kg OLE, followed by a reduction in the group that received 2 g/kg OLE. Fazio et al. [40] observed a significant increase in serum globulin, albumin, and total protein levels in fish fed 1.5 and 2% OLE. Naz et al. [26] observed that fish fed diets of 1.5 and 2% OLE had significantly higher levels of serum total protein, albumin, and globulin. Baba et al. [63] reported an increase in serum total protein levels in fish fed OLE-containing diets, with a peak at dose of 0.5%. This dose-dependent increase of up to 1 g/kg OLE in our results may be associated with stimulation of the immune response, which reflects a higher production of transport and acute-phase proteins. The reduction in total plasma protein observed in fish fed a diet with 2 g/kg OLE may have been induced by oxidative stress. This condition can alter gene expression related to energy metabolism, immune and antioxidant enzymes, and other cellular defense proteins [77]. An increase in total protein has been commonly observed in fish fed diets containing leaf extracts and is often associated with greater resistance to infections and an improved innate immune response, attributed to the action of the polyphenols present in these extracts [17,26,41,78]. Hoseini et al. [41] observed an increase in total protein and plasma globulin levels at the lowest inclusion level (1%) of olive leaf extract (*Elaeagnus angustifolia*). Zemheri-Navruz et al. [44] observed that immune-related variables, including total protein, decreased significantly in fish fed high doses of OLE. In our study, the maintenance of total hepatic protein levels in fish was neither compromised nor significantly stimulated.

Lipid oxidation causes the degradation of unsaturated fatty acids, which generates several oxidation products, such as peroxides, aldehydes, and ketones, which not only reduce the organoleptic quality of aquatic products, but also degrade their nutrients, reducing their nutritional value as a whole [79]. Malondialdehyde (MDA) is recognized as the main product of lipid peroxidation. Compared to free radicals, MDA has a relatively long half-life and an uncharged structure [79]. When the antioxidant defense mechanism is insufficient, free radical levels increase, resulting in increased lipid oxidation, oxidative damage to membranes, and eventual disturbance of essential cellular activities [60]. In our study, lipid peroxidation in fish muscle did not show any alterations. However, the fish that received the lowest dose of OLE in the diet (0.25 g/kg) had the lowest level of lipid peroxidation in the liver. This result may be related to the antioxidant activity of oleuropein, the main bioactive compound present in OLE. González-Ortega et al. [80] demonstrated that oleuropein has a significant ability to sequester free radicals and inhibit lipid peroxidation. Complementary studies have corroborated this finding. Although we did not directly evaluate antioxidant enzyme activity in our study, Buzdar et al. [81] observed that OLE supplementation (300 mg/kg) increased the activity of antioxidant enzymes, such as superoxide dismutase (SOD), catalase (CAT) and glutathione (GSH), in rat liver tissues, in addition to reducing the production of MDA, evidencing its efficacy in protecting against oxidative stress and preserving cellular integrity. Oleuropein has also been shown to reduce cytokine-induced generation of reactive oxygen species (ROS) and restore the activity of the antioxidant system [60]. A significant reduction in lipid peroxidation was observed in diabetic rats treated with oleuropein [60]. The antioxidant capacity of oleuropein is also related to its ability to chelate metal ions such as iron and copper, which participate in ROS generation through Fenton reactions.

## 5. Conclusions

The results of the present study indicate that the inclusion of olive leaf extract (OLE) in the diet of juvenile Nile tilapia does not compromise growth parameters or zootechnical indices. In addition, the 0.25 g/kg dose demonstrated beneficial effects, improving condition factors, body composition, and health-related biochemical parameters, as well as reducing hepatic lipid peroxidation. Future studies should investigate the physiological mechanisms involved, as well as evaluate different exposure times to OLE, to optimize its use in fish nutrition.

## Figures and Tables

**Table 1:** Formulation and centesimal composition of the diets used during the experimental trial.

Ingredients (g/kg)	OLE_0_	OLE_0.25_	OLE_0.5_	OLE_1.0_	OLE_2.0_
Fishmeal ^1^	300	300	300	300	300
Gelatin ^2^	30	30	30	30	30
Soybean meal ^3^	200	200	200	200	200
Corn starch ^4^	347.5	347.5	347.5	347.5	347.5
Soybean oil ^5^	12.5	12.5	12.5	12.5	12.5
Wheat bran ^6^	100	100	100	100	100
Mineral and vitamin premix ^7^	10	10	10	10	10
OLE	0	0.25	0.5	1	2
Moisture (%)	3.37 ± 0.09	2.56 ± 0.15	2.45 ± 0.20	3.42 ± 0.26	3.31 ± 0.15
Ash (%)	9.47 ± 0.14	9.44 ± 0.09	9.46 ± 0.03	9.26 ± 0.16	9.37 ± 0.06
Crude protein (%)	33.65 ± 0.25	33.50 ± 0.11	33.59 ± 0.48	33.71 ± 0.35	33.23 ± 0.13
Total lipid (%)	7.68 ± 0.29	7.84 ± 0.53	8.49 ± 0.81	8.21 ± 0.42	8.29 ± 0.31
Crude fiber (%)	1.94 ± 0.10	1.45 ± 0.21	2.16 ± 0.77	1.91 ± 0.22	1.53 ± 0.10
NNE	43.89	45.21	43.85	43.49	44.27

^1^ PETFAR, Nova Bréscia, RS, Brazil; ^2^ Dinamica Quimi. Contemp. Ltda, RS, Brazil; ^3^ Sulino, RS, Brazil; ^4^ Tok, Vogel Alimentos, Canoas, RS, Brazil; ^5^ Corcovado, ADM do Brasil Ltda., Brazil; ^6^ MOTASA, Taquari, RS, Brazil; ^7^ Tectron, Toledo, PR, Brazil; Vitamin A (min.) 2000 UI/kg, vitamin D3 (min.) 640.000 UI/kg, vitamin E (min.) 2.400 UI/kg, vitamin K3 (min.) 680 mg/kg, vitamin B1 (min.) 400 mg/kg, vitamin B2 (min.) 1.000 mg/kg, vitamin B6 (min.) 1.200 mg/kg, vitamin B12 (min.) 4.000 mcg/kg, niacin (min.) 9.000 mg/kg, pantothenic acid (min.) 3.000 mg/kg, folic acid (min.) 400 mg/kg, biotin (min.) 35 mg/kg, manganese (min.) 14 g/kg, zinc (min.) 11 g/kg, iron (min.) 10 g/kg, copper (min.) 2.000 mg/kg, iodine (min.) 200 mg/kg, cobalt (min.) 40 mg/kg, selenium (min.) 40 mg/kg.

**Table 2:** Growth parameters of Nile tilapia juveniles fed the experimental diets.

Parameters	Treatments				
	OLE_0_	OLE_0.25_	OLE_0.5_	OLE_1.0_	OLE_2.0_
IW (g)	0.55 ± 0.11	0.55 ± 0.10	0.55 ± 0.10	0.55 ± 0.10	0.55 ± 0.10
FW (g)	21.02 ± 0.85	20.60 ± 1.35	19.41 ± 1.78	21.41 ± 1.54	20.61 ± 0.91
WG (g)	20.47 ± 0.85	20.04 ± 1.35	18.85 ± 1.78	20.85 ± 1.54	20.05 ± 0.90
DWG (g)	0.44 ± 0.01	0.43 ± 0.02	0.40 ± 0.03	0.45 ± 0.03	0.43 ± 0.01
SGR (%)	7.88 ± 0.08	7.83 ± 0.13	7.70 ± 0.19	7.91 ± 0.15	7.84 ± 0.08
SR (%)	100 ± 0	100 ± 0	100 ± 0	100 ± 0	100 ± 0
FCR	1.03 ± 0.07	1.06 ± 0.03	1.08 ± 0.02	1.01 ± 0.04	1.10 ± 0.03
CF	2.58 ± 0.46 ^ab^	2.64 ± 0.52 ^a^	2.44 ± 0.47 ^ab^	2.46 ± 0.41 ^ab^	2.33 ± 0.48 ^b^

The values in the table are described as mean ± standard deviation. Values in the same row with different letters indicate significant differences between treatments (*p* < 0.05). IW = initial weight; FW = final weight; WG = weight gain; DWG = daily weight gain; SGR = specific growth rate; SR = survival rate; FCR = feed conversion ratio; CF = condition factor.

**Table 3:** Zootechnical indices of Nile tilapia juveniles fed the experimental diets.

Parameters	Treatments				
	OLE_0_	OLE_0.25_	OLE_0.5_	OLE_1.0_	OLE_2.0_
HSI (%)	1.01 ± 0.18	1.10 ± 0.35	1.05 ± 0.43	1.12 ± 0.29	0.96 ± 0.32
VSI (%)	5.09 ± 0.95	5.81 ± 1.27	5.90 ± 1.50	6.04 ± 0.78	6.37 ± 1.18
CY (%)	85.42 ± 0.98	85.62 ± 0.78	85.33 ± 1.35	84.92 ± 1.40	85.61 ± 1.27
IQ	6.63 ± 1.67	6.33 ± 1.53	5.67 ± 1.23	5.64 ± 1.87	6.17 ± 1.10

The values in the table are described as mean ± standard deviation. HSI = hepatosomatic index; VSI = viscerosomatic index; CY = carcass yield; IQ = intestinal quocient.

**Table 4:** Centesimal composition of the whole body (wet basis) and nutrient deposition of Nile tilapia juveniles fed the experimental diets.

Parameters		Treatments				
	Initial	OLE_0_	OLE_0.25_	OLE_0.5_	OLE_1.0_	OLE_2.0_
Moisture (%)	81.43 ± 0.01	71.95 ± 0.58 ^a^	70.20 ± 0.55 ^b^	71.00 ± 0.47 ^ab^	71.25 ± 1.34 ^ab^	71.66 ± 0.81 ^a^
Ash (%)	3.82 ± 0.01	3.96 ± 0.31	3.93 ± 0.11	3.95 ± 0.14	3.90 ± 0.09	4.05 ± 0.22
Crude protein (%)	12.84 ± 0.04	14.94 ± 0.39 ^b^	15.73 ± 0.15 ^a^	15.65 ± 0.64 ^a^	15.64 ± 0.43 ^a^	15.48 ± 0.44 ^ab^
Total lipid (%)	2.16 ± 0.18	7.54 ± 0.68 ^c^	8.99 ± 0.48 ^a^	8.11 ± 0.31 ^bc^	8.28 ± 0.77 ^b^	7.53 ± 0.23 ^c^
Protein deposition (g)	-	3.06 ± 0.12	3.17 ± 0.20	2.95 ± 0.14	3.27 ± 0.14	3.12 ± 0.21
Lipid deposition (g)	-	1.57 ± 0.22	1.84 ± 0.12	1.56 ± 0.18	1.75 ± 0.06	1.54 ± 0.08

The values in the table are described as mean ± standard deviation (*n* = 9). Values in the same row with different letters indicate significant differences between treatments (*p* < 0.05).

**Table 5:** Biochemical parameters of Nile tilapia juveniles fed the experimental diets.

Parameters	Treatments				
	OLE_0_	OLE_0.25_	OLE_0.5_	OLE_1.0_	OLE_2.0_
Muscle glycogen (mg/g)	0.78 ± 0.22 ^a^	0.54 ± 0.12 ^b^	0.60 ± 0.18 ^b^	0.59 ± 0.12 ^b^	0.62 ± 0.11 ^b^
Hepatic glycogen (mg/g)	0.71 ± 0.30 ^abc^	0.57 ± 0.43 ^c^	0.97 ± 0.62 ^a^	0.67 ± 0.31 ^b^	0.61 ± 0.37 ^b^
Plasma glucose (mg/dL)	78.51 ± 14.57	81.88 ± 10.65	76.69 ± 11.98	76.23 ± 15.48	76.34 ± 12.49
Plasma cholesterol (mg/dL)	161.11 ± 12.89	159.47 ± 16.01	153.84 ± 20.20	155.72 ± 15.00	152.44 ± 15.10
Plasma triglycerides (mg/dL)	141.10 ± 7.72 ^abc^	141.01 ± 8.02 ^bc^	136.30 ± 6.01 ^c^	147.58 ± 14.80 ^a^	144.93 ± 10.09 ^ab^
Total plasma protein (g/dL)	3.16 ± 0.22 ^ab^	3.14 ± 0.23 ^ab^	3.10 ± 0.18 ^ab^	3.21 ± 0.21 ^a^	2.98 ± 0.25 ^b^
Hepatic glucose (mg/g)	8.68 ± 3.80 ^b^	10.11 ± 2.78 ^b^	9.35 ± 3.55 ^b^	10.32 ± 5.32 ^b^	13.86 ± 4.43 ^a^
Hepatic cholesterol (mg/g)	4.91 ± 0.45	5.03 ± 0.41	5.16 ± 0.33	4.71 ± 0.86	4.91 ± 0.73
Hepatic triglycerides (mg/g)	28.46 ± 6.06 ^ab^	26.23 ± 7.05 ^b^	30.69 ± 6.86 ^a^	25.33 ± 9.21 ^ab^	28.59 ± 8.64 ^ab^
Total hepatic protein (g/g)	2.04 ± 0.50	2.00 ± 0.47	1.93 ± 0.45	2.33 ± 0.79	2.25 ± 0.54

The values in the table are described as mean ± standard deviation (*n* = 9). Values in the same row with different letters indicate significant differences between treatments (*p* < 0.05).

## Data Availability

The data presented in this study are available on request from the corresponding author.
